# A guide for planning triangulation studies to investigate complex causal questions in behavioural and psychiatric research

**DOI:** 10.1017/S2045796024000623

**Published:** 2024-11-07

**Authors:** Jorien L Treur, Eva Lukas, Hannah M Sallis, Robyn E Wootton

**Affiliations:** Genetic Epidemiology, Department of Psychiatry, Amsterdam UMC, University of Amsterdam, the Netherlands; Genetic Epidemiology, Department of Psychiatry, Amsterdam UMC, University of Amsterdam, the Netherlands; Centre for Academic Mental Health University of Bristol, Bristol, UK; Department of Population Health Sciences, Bristol Medical School, University of Bristol, Bristol, UK; Medical Research Council (MRC) Integrative Epidemiology Unit, University of Bristol, Bristol, UK; Nic Waals Institute, Lovisenberg Diaconal Hospital, Oslo, Norway; MRC Integrative Epidemiology Unit, University of Bristol, Bristol, UK; Population Health Sciences, Bristol Medical School, University of Bristol, Bristol, UK; School of Psychological Science, University of Bristol, Bristol, UK

**Keywords:** Triangulation, Causality, Bias, Prevention, Behaviour, Psychiatry

## Abstract

**Aims:**

At the basis of many important research questions is causality – does X causally impact Y? For behavioural and psychiatric traits, answering such questions can be particularly challenging, as they are highly complex and multifactorial. ‘Triangulation’ refers to prospectively choosing, conducting and integrating several methods to investigate a specific causal question. If different methods, with different sources of bias, all indicate a causal effect, the finding is much less likely to be spurious. While triangulation can be a powerful approach, its interpretation differs across (sub)fields and there are no formal guidelines. Here, we aim to provide clarity and guidance around the process of triangulation for behavioural and psychiatric epidemiology, so that results of existing triangulation studies can be better interpreted, and new triangulation studies better designed.

**Methods:**

We first introduce the concept of triangulation and how it is applied in epidemiological investigations of behavioural and psychiatric traits. Next, we put forth a systematic step-by-step guide, that can be used to design a triangulation study (accompanied by a worked example). Finally, we provide important general recommendations for future studies.

**Results:**

While the literature contains varying interpretations, triangulation generally refers to an investigation that assesses the robustness of a potential causal finding by explicitly combining different approaches. This may include multiple types of statistical methods, the same method applied in multiple samples, or multiple different measurements of the variable(s) of interest. In behavioural and psychiatric epidemiology, triangulation commonly includes prospective cohort studies, natural experiments, and/or genetically informative designs (including the increasingly popular method of Mendelian randomization). The guide that we propose aids the planning and interpreting of triangulation by prompting crucial considerations. Broadly, its steps are; determine your causal question, draw a directed acyclic graph, identify available resources and samples, identify suitable methodological approaches, further specify the causal question for each method, explicate the effects of potential biases, and pre-specify expected results. We illustrated the guide’s use by considering the question: “Does maternal tobacco smoking during pregnancy cause offspring depression?”.

**Conclusions:**

In the current era of big data, and with increasing (public) availability of large-scale datasets, triangulation will become increasingly relevant in identifying robust risk factors for adverse mental health outcomes. Our hope is that this review and guide will provide clarity and direction, as well as stimulate more researchers to apply triangulation to causal questions around behavioural and psychiatric traits.

## Introduction

**Figure 1:**
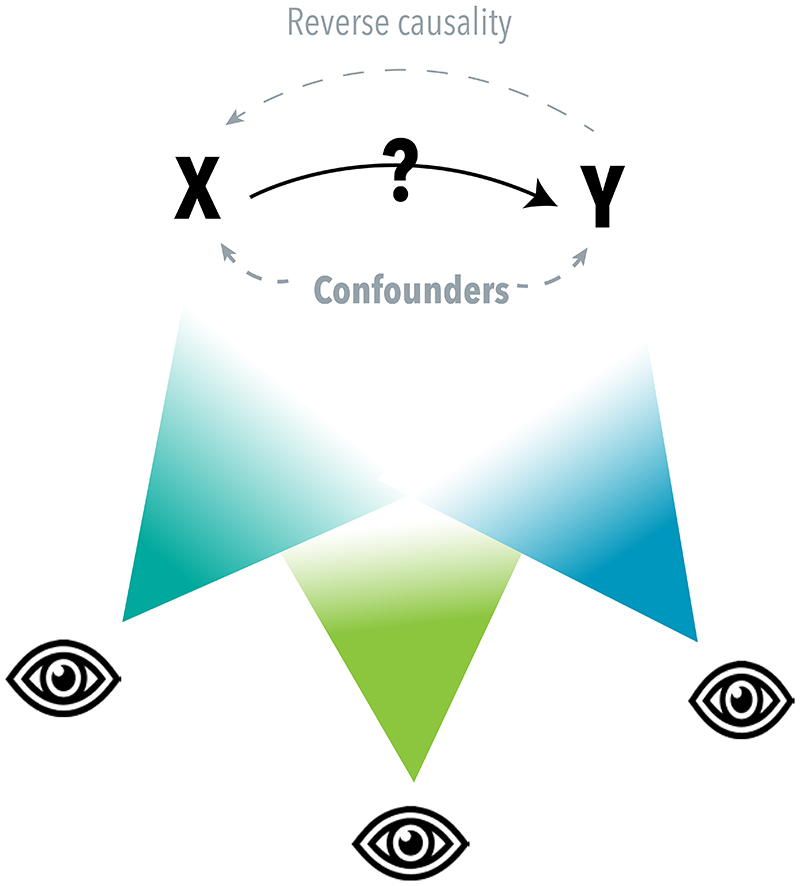
The concept of triangulation. When two variables of interest, X and Y, are associated, this may be due to a non-causal explanation (i.e., confounding or reverse causality) or a causal explanation (X causally affecting Y). Triangulation can be seen as a process that uses different ‘points of view’ to come to a more reliable answer to a causal question. It refers to the use of different approaches, with different underlying biases, strengths, and/or weaknesses, to assess the same causal question. It is important to clarify that the goal of triangulation is not solely to pinpoint a precise point estimate of the causal effect, but rather to gather more reliable evidence regarding the direction and nature of the effect. Common types of triangulation are the use of different analytical methods, applying the same method across different study samples, and/or using different types of measures. All of these are meant to obtain more robust evidence on causality, which can help determine whether or not an intervention or change in X could beneficially impact Y.

**Figure 2:**
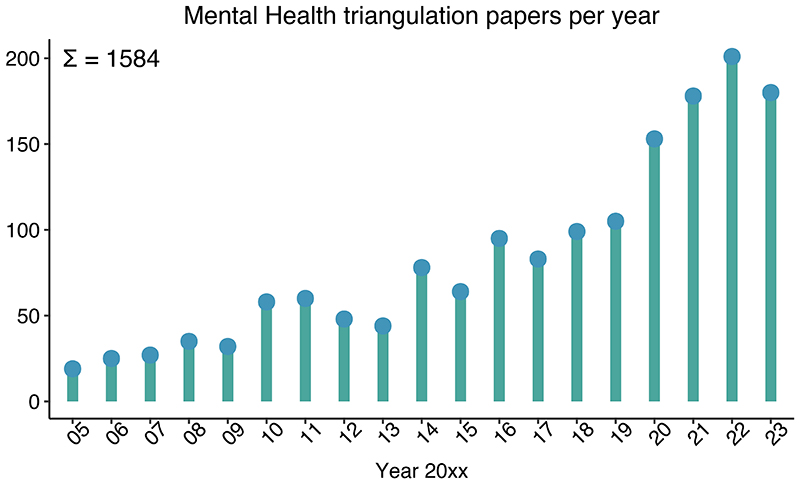
Accumulation of research papers mentioning or facilitating ‘triangulation’ The number of publications in mental health and substance abuse research has increasingly gained in popularity in recent years. We show the significant trend of an increased number of scientific outputs, resulting from our PubMed search strategy: 2005/01/01:2023/12/31[Date - Publication] AND “triangulation”[Title/Abstract] AND (“mental *”[Title/Abstract] OR “schizophrenia”[Title/Abstract] OR “depression”[Title/Abstract] OR “anxiety”[Title/Abstract] OR “bipolar”[Title/Abstract] OR “suicid*”[Title/Abstract] OR “ADHD”[Title/Abstract] OR “Autism”[Title/Abstract] OR “behaviour*”[Title/Abstract] OR “behavior*”[Title/Abstract] OR “psycholog*”[Title/Abstract]). Since 2005, 1584 ‘triangulation’ studies have been published in the field. This upward trend underscores the need for a more systematic guide for triangulation efforts and harmonize diverse approaches.

At the basis of many important research questions is causality – does X causally impact Y? Answering such questions is particularly challenging for behavioural and psychiatric traits, as they are highly complex and multifactorial in their aetiology. Most of the time, conducting a randomized trial, the ‘gold standard’ of causality testing^9^, is not feasible due to practical and/or ethical restrictions. Instead, researchers try to answer complex causal questions by using other methods that may help determine if changes in one variable lead to changes in another. Examples of such methods are longitudinal cohort analyses corrected for relevant confounders or natural experiments that leverage the effects of a policy change or genetic variants.^10^ Any such individual method relies on particular assumptions and has certain limitations. In order to overcome the weaknesses of individual methods, ‘*triangulation*’ of several methods can be applied. The idea here is that *prospectively* choosing and then conducting several methods with different (preferably uncorrelated) biases will lead to more reliable evidence and ultimately more robust conclusions.^11^ If different methods, with different sources of bias, all indicate a causal effect in the same direction, the finding is less likely to be spurious (depicted as different ‘points of view’ on a causal question in Figure 1).^11^ Triangulation is not a new concept, but its application has rapidly gained popularity in behavioural and psychiatric epidemiology in recent years (Figure 2). While triangulation has been eloquently described by others^12,13^ a more ‘hands-on’ guide with clear steps and considerations that can be used by researchers who are planning to conduct a triangulation study is currently not available. Here, we would like to provide more clarity and guidance around the process of triangulation, so that new triangulation studies can be better designed and results can be better interpreted. Ultimately, this will help researchers to determine which associations are causal and, consequently, which variables can be used to improve prevention and treatment of complex mental health problems.

Specifically, we aim to: 1) explain the concept of triangulation and how it is applied in behavioural and psychiatric epidemiology, 2) put forth a step-by-step guide that can be used to design a triangulation study, including a worked example, and 3), provide important general recommendations.

### 1 The concept of triangulation

#### What is triangulation?

The term triangulation originates from an approach that determines the location of a point of interest by measuring angles to it from two or more (known) points and applying trigonometry (i.e., using the properties of triangles).^11^ In scientific research, usually triangulation is interpreted as conducting two or more research methods to obtain the answer to a complex question, although it is used differently across different research fields (see Box 1). By synthesizing multiple sources of information, the overall evidence is assumed to be more reliable, and more robust conclusions can be derived. The idea, that if a finding replicates across different settings, it is more likely to be true, is also incorporated in the well-known Bradford-Hill guidelines for causality as the ‘consistency’ criterion.^14^ To some degree, it lies at the basis of scientific practice in general. Researchers usually compare findings of their empirical work to those from other studies in the discussion section of a paper or in a review, acknowledging strengths and weaknesses. Systematic reviews and particularly meta-analyses aim to *retrospectively* combine estimates from similar analyses to come to a more precise point estimate.^15^ If these estimates come from the same method, some error might be removed by combining across them, but consistent bias inherent to the method will remain.

Box 1: Triangulation across research fields

Triangulation has been used across various research fields, including epidemiology, but also education and sociology.^1–4^ In the 80’s, sociologist Norman Denzin posed that a body of various methods represents different aspects of reality, each with their own weight and relevance to the observed phenomena; *“If each method leads to different features of empirical reality, then no single method can ever completely capture all the relevant features of that reality; consequently, sociologists must learn to employ multiple methods in the analysis of the same empirical events*”.^5^ In sociology in particular, triangulation is often interpreted as “mixed-method research”, i.e., the combination of quantitative and qualitative methods.^4,6^ There has been criticism on this specific application of triangulation. For instance, Erzberger and Prein (1997) state that measures of the same variable of interest with such different (quantitative versus qualitative) procedures may not reflect the same “shape of reality”.^7^ Within the realm of quantitative methods, similar points of criticism could be made, such that results from (very) different data sources may be incompatible and therefore challenging to interpret together.^8^ As applied in more recent literature, quantitative triangulation is typically used to test the existence and direction of a potential causal effect, but not necessarily to estimate the precise effect size.

#### Triangulation in the field of behavioural and psychiatric epidemiology

Here, we focus on the use of triangulation to answer aetiological questions about behavioural and psychiatric traits. In this context, a precise definition of triangulation has been proposed by Lawlor, Tilling, and Davey Smith (2016) as: “*The practice of strengthening causal inferences by integrating results from several different approaches, where each approach has different (and assumed to be largely unrelated) key sources of potential bias*.” Differing from *retrospective* synthesis of evidence (e.g. systematic reviews), the definition of triangulation is predominantly *prospective*.^11,13^ It entails deliberately selecting several approaches to complement one another, conducting them in unison and then synthesizing and interpreting the results together. Approaches can vary in 1) the methods applied, 2) the population from which the sample is drawn, and/or 3) the measurement of the variables.

#### Approach 1: Triangulation using different methods

It is recommended that two or more methods with different sources of potential bias should be used to address the same underlying causal question.^11^ Triangulation is most effective if the selected methods have different expected directions of bias. This means that where the bias of one method may lead to an overestimation of the true causal effect, the bias of another method may be towards the null (or even towards the opposite sign of the effect).^11^ In Supplementary Table 1, we provide a broad, but non-exhaustive overview of methods that may be used in triangulation studies, meant to serve as an inspiration for introducing breadth. Under methodological triangulation, we differentiate experimental from observational studies. Experimental studies in the form of randomized trials are ideal for causal inference, but usually unfeasible in behavioural and psychiatric epidemiology. This is largely due to practical and ethical restrictions, but also the long timespan that it would take for a potential causal effect to arise. In some instances, however, experimental manipulation can be used to assess relevant short-term processes that may tell you something about longer-term effects. In observational research, there is a wide range of methods that try to determine whether one variable affects another (although this causal goal is not always acknowledged^16^), the most common being analysis of longitudinal cohort data to test if changes in the exposure are followed by changes in the outcome. Certain observational methods can be considered ‘quasi-experimental’ because some type of (environmental) influence or change is leveraged to create quasi-experimental groups in a given population, e.g. a policy change.^17,18^ Current triangulation literature also incorporates many relatively novel causal methods from the field of genetic epidemiology, which has leveraged major technical advances in molecular genetics over the past two decades.^19^ The most notable is Mendelian randomization (MR), which uses genetic variants as instrumental variables to proxy for levels of the exposure.^20^ Other genetically informative designs don’t use molecular genetic data, but instead rely on inferences based on familial relatedness, such as discordant twin and sibling-control studies.^21^

An example of methodological triangulation by Van de Weijer and colleagues (2023) explored whether spending more years in education causally increases later-life mental wellbeing.^22^ They compared results across four designs: 1) a natural experiment using the raising of the school leaving age policy change 2) a sibling-control design which accounts for shared familial confounding, 3) MR, and 4) within-family MR, which further accounts for assortative mating, population stratification and dynastic effects.^23^ An additional example of methodological triangulation comes from Esen and colleagues (2022). Seeking to understand whether in utero exposure to anti-depressant drugs causes offspring ADHD, Esen and colleagues (2022) compared results from four designs: 1) regular analysis controlling for confounders, 2) a negative-control analysis comparing the association of paternal antidepressant use with that of pregnant mothers, 3) sibling-control analysis comparing siblings who were discordant for maternal antidepressant use during pregnancy and 4) a former-user analysis comparing with mothers who previously took antidepressants but not during pregnancy, to mitigate confounding via maternal depression status.^24^ For both of the triangulation examples here, the authors purposefully selected designs that each help correct for different sources of bias, where converging evidence across the different methods would suggest a true causal effect.

#### Approach 2: Triangulation using samples from different populations

Second, let us consider triangulating across samples from different populations. This form of triangulation can help to mitigate bias from confounding and selection pressures. An overview of applications of this approach is given in Supplementary Table 1. A particularly illustrative example comes from Brion et al. (2011), who tested the (potentially causal) effect of breastfeeding on cognitive development in offspring. Breastfeeding is strongly associated with socio-economic position (SEP) which is also a major confounder for the breastfeeding-cognitive development relationship. Conveniently, the direction of the confounding effect of SEP is different in the United Kingdom, where breastfeeding is associated with higher SEP, than in Brazil, where breastfeeding is associated with lower SEP. This makes the underlying confounding structures of the samples different. By investigating the causal question if breastfeeding positively affects cognitive development in both samples, confounding by SEP as an explanation can be partly circumvented and finding a causal effect in both samples would constitute more reliable evidence overall.^25^

As an additional example, Chartier and colleagues (2023) explored whether immigration increases risk for alcohol use disorder (AUD) through acculturation. Acculturation refers to the assimilation of the less dominant into the more dominant culture.^26^ Chartier and colleagues (2023) hypothesize that the rates of AUD in the immigrant populations will become more similar to the rates in the native population over subsequent generations. They compared immigrants in Sweden from seven different geographical regions, each with different starting levels of AUD. For example, 1^st^ generation immigrants from Africa have lower rates of AUD than the Swedish population and Finnish 1^st^ generation immigrants have higher rates. Over subsequent generations, rates of AUD in both populations became closer to the Swedish average. Change in different directions makes this less likely to be due to confounding and more likely due to acculturation.

#### Approach 3: Triangulation using different measures

Finally, triangulation could compare different types of measures for the variable of interest, for instance taken from different sources, raters or obtained with different measurement instruments. For example, while self-report can be affected by recall or social desirability bias, registry data may be measured with error due to missingness or incorrectly recorded disease codes whilst obtaining a correct diagnosis. An illustrative example comes from Haan et al (2021) who explored the association between foetal alcohol exposure and ADHD risk in offspring.^27^ They observed different patterns of association depending on whether ADHD symptoms were mother or teacher reported, and depending on the scale used. With no single measure being free from bias, triangulating across several available measures can help strengthen conclusions. However, careful consideration of the interpretation is needed when results differ. An overview of how this approach could be applied is given in Supplementary Table 1.

### 2 A step-by-step guide for designing a triangulation study

**Figure 3:**
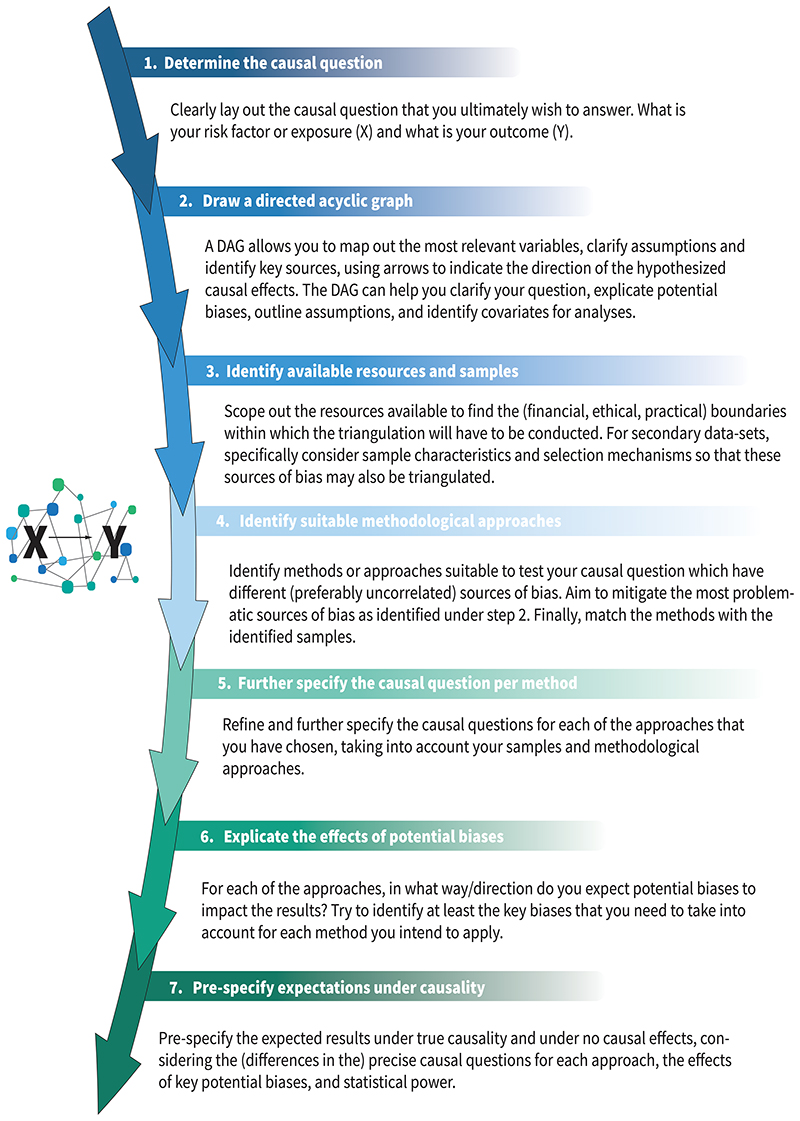
Triangulation guide. This step-by-step guide is meant to take researchers along through the most important steps of designing a triangulation study, highlighting the most important considerations and current best practices.

A successful triangulation study could take many forms, depending upon the causal question and available data, but should always follow the key principle of triangulation – that more powerful causal conclusions are drawn by combining multiple approaches with different sources of bias. In this section, we provide a step-by-step guide to aid the planning and interpretation of triangulation studies (Figure 3). Our guide doesn’t aim to be prescriptive nor exhaustive, but rather to prompt the crucial considerations necessary for an effective triangulation protocol. We accompany this with a worked example, where we work through each step of the guide (see Supplementary Note).

#### Step 1: Determine the causal question

It is of upmost importance to begin with a clear causal research question. The best causal questions are informed by theory and existing literature and take into account whether or not a potential effect is restricted to a particular timing or length of exposure. For our worked example, we ask the causal question ‘Does maternal tobacco smoking during pregnancy cause offspring depression?’.^28^ It should be noted that the initial question that is posed at the start of a triangulation study, will translate to (slightly) different, but related, questions across the different approaches that are subsequently chosen (see step 5 of this guide). Approaches which are testing more similar questions will be easier to integrate and interpret together. Ultimately, triangulation aims to come to the best-fitting explanation for an observed relationship, based on the totality of the data.

#### Step 2: Draw a directed acyclic graph

Directed acyclic graphs (DAGs) can be used as a tool for mapping out assumed relationships between relevant variables.^29^ In essence, a DAG is a graph that consists of the variables of interest with arrows between the variables indicating the direction of hypothesized causal effects and a lack of an arrow between any two variables implying that there is no hypothesized causal effect. A DAG is ‘acyclic’, meaning that cycles are not allowed. In the context of triangulation, DAGs can help you clarify your assumptions, establish key sources of bias, and identify appropriate covariates for statistical analysis. A DAG for our worked example is given in the Supplementary Note.

#### Step 3: Identify available resources and samples

With your causal question defined, the next step is to scope out the resources available. Here, the aim is to find the boundaries within which the triangulation will have to be conducted, which may represent financial, ethical or practical constraints. For many causal questions in aetiological epidemiology, there will not be sufficient resources or time to collect new data. Therefore, identification of suitable secondary data often presents the most effective way to conduct a triangulation study. When identifying possible datasets, consideration should be given to sample characteristics and selection mechanisms, so that these sources of bias can also be triangulated.

Let us take our worked example, does maternal tobacco smoking during pregnancy cause offspring depression? The timing of our question is specific to intrauterine effects. Therefore, suitable datasets are restricted to those with relevant smoking data during pregnancy and long enough follow-up to capture depression traits in offspring. As collecting new prospective longitudinal data to investigate this question would be too time-consuming and expensive,^24^ we identify only relevant secondary data sources for this worked example (see Supplementary Note).

#### Step 4: Identify suitable methodological approaches

At this stage, we identify methods which are suitable to test the causal question and have different (preferably uncorrelated) sources of bias. The aim here, is to try and mitigate the most problematic sources of bias as identified in step 2. Here, we identify which methods are feasible given the available samples.

In the Supplementary Note, we expand upon five methodological approaches that would allow us to test whether there is evidence for causal effects of maternal smoking during pregnancy on offspring depression. From Supplementary Table 1, several methods were not suitable, for example, any type of randomisation to smoking during pregnancy would pose serious ethical concerns. However, there were still a multitude of study designs which could help investigate our causal question, within the scope of available resources. First, to address potential confounding by environmental factors (e.g. SEP) and by mother’s own depression, we propose a longitudinal regression model with adjustment for confounding in an existing prospective pregnancy cohort. This method can still be biased by residual confounding and doesn’t account for shared genetic liability between parents and offspring. In other words, if the mother is more likely to smoke, the offspring is more likely to smoke and their own smoking could cause their depression. To address these biases, we would plan to conduct an intergenerational MR study. This approach uses genetic variants as instrumental variables to reduce bias from residual confounding, and adjusts for offspring genotype to isolate maternal effects.^30^ Additionally, we would add a proxy GxE MR study, which tests whether maternal smoking during pregnancy is associated with offspring depression. If we observe an association between the genetic variants for maternal smoking and offspring depression, regardless of maternal smoking status, effects are likely non-specific to smoking during pregnancy. Finally, we would repeat the confounder adjustment and MR analyses with father’s smoking as a powerful negative control (since the father cannot have direct intrauterine effects on the offspring).

Statistical power is an important factor to consider here, because it will determine the likelihood for each method to pick up on a true causal effect. For new data collection, conducting an a priori power calculation is important and highly recommended. For secondary data analysis (where data collection has already occurred) there is no consensus about the usefulness of conducting power analyses.^31^ Where possible (i.e., if the data are already accessible), we consider a power analysis at this stage beneficial, as it can be taken into account when specifying your expectations of potential findings and how to interpret them. When key information to conduct a power analysis is not available, it may be computed post-hoc to aid interpretation. While underpowered analyses should generally be avoided there are certainly cases where adding an analysis that could mitigate an important source of bias would be valuable.

#### Step 5: Further specify the causal question per method

After having posed the initial causal question under step 1, here, we aim to define the specific questions that can be answered with each of the approaches that we have chosen. To facilitate the integration of findings from different approaches, we recommend researchers formulate their causal questions as specifically as possible in this step. Drawing specific DAGs for each approach may be useful during this refinement process. To illustrate, the specific causal question for the confounder adjusted analysis in the ALSPAC cohort (see Supplementary Note) is: ‘In a sample of offspring born in the early 90s in Avon (UK), is mother-reported maternal tobacco smoking during pregnancy (in any trimester) associated with offspring-reported offspring depressive symptoms up to age 30 years, after adjustment for measured confounders?’

#### Step 6: Explicate the effects of potential biases

Once the most suitable methods, samples and measures for triangulation have been chosen, and specific questions have been formulated, it is important to explicate the potential effects of biases. Of course, it is not possible for the effects of all potential biases to be known, by their very definition some are unknown (e.g., residual confounding). In Supplementary Table 1 we highlight the most notable sources of bias for a selection of approaches.

#### Step 7: Pre-specify expectations under causality

For triangulation to be most effective, it is important to specify a priori what results we would expect to see under true causality and what results we may expect if our association of interest is in fact not causal.^32^ Most often, pre-specification of a true causal effect is straightforward – we would expect each of the methods to show statistical evidence for an effect in the same direction across the methods/cohorts/samples fulfilling broadly similar characteristics (such as age). When specifying expected causal effects, it is important to consider the power of each method. For example, Van de Weijer and colleagues (2023) pre-specified that they would not expect the within-family MR results to pass the threshold for statistical significance given low power and that consequently, for that method they would be looking to see whether the direction of effect was consistent with the other methods.^22^

Another important consideration is the timing and duration of the exposure of interest across different methods. If for one method the influence of the exposure is relatively short (for instance in an experimental setting) while in another method it is relatively long (for instance in a MR study where the genetic variants express a lifetime influence), it is to be expected that the size of the effect would differ. This needs to be taken into consideration when pre-specifying your expectations, and later, when comparing the results. If one method finds evidence for a causal effect and another doesn’t, but this can convincingly be explained based on (pre-specified) differences in the timing, measurement, or duration of the exposure, it might not decrease the overall evidence for causality.

More difficult, but equally as important, is to pre-specify your expected results under a non-causal model. Where known (or deducible), it is useful to begin by pre-specifying the expected biases of each method, and their expected direction of effect. This can help to ensure that you have selected methods with uncorrelated biases. For example, Van de Weijer and colleagues (2023) specified a priori that they would conclude that there was substantial confounding or bias from reverse causation if results were not significant and consistent.^22^ When defining such expectations, it is important to consider your limitations with regard to statistical power. Identifying true causal relationships requires the presence of robust outcomes with strong statistical evidence. If your analyses are underpowered then small effects might not be detected. Nonetheless, it is likely that utilizing diverse methodologies across potentially heterogeneous samples leads to outcomes of differing levels of (statistical) strength. To employ an analogy, envision a reservoir: with each piece of evidence stemming from a method or sample, water is added, its volume proportional to the method’s significance and weight. Conversely, opposing effects would deplete the reservoir’s reserves. The overall evidence for causality is reflected in the fullness of the reservoir.

### 3 General recommendations for future triangulation studies

In addition to our step-by-step guide, here we would like to highlight some important, more general recommendations for those planning to conduct a triangulation study.

#### Triangulation in the context of the broader literature

A well-designed triangulation study should be clearly informed by the existing literature and set up to address the most important limitations of prior studies. The results of a triangulation study should also be evaluated within the context of the body of evidence that already exists, taking into account the inherent biases of each line of evidence and their capacity to reflect reality. If we want to come to more reliable scientific evidence to answer complex causal questions in behavioural and psychiatric epidemiology, it is crucial to acknowledge which exact biases are relevant for which methods, samples, and measures, both when we compare the results of analyses within one (triangulation) study as well as when we compare results across studies.

#### Falsification

One approach that can be particularly powerful in a triangulation framework but which is not applied widely enough, is the negative-control analysis. In the context of the worked example in this review, we chose a negative-control design where we compared the effect of maternal smoking during pregnancy on offspring depression to the effect of paternal smoking during pregnancy. Father’s smoking cannot have direct causal intrauterine effects on the offspring, therefore if paternal effects have a similar magnitude of association as maternal effects, this is likely due to bias from confounding. We recommend researchers to adapt an ethos of attempting to falsify a potentially causal finding, for which negative-control analysis is very suitable. Note that either a negative-control outcome variable or a negative-control exposure variable can be tested. It is important to select valid negative controls, where it is clear or well-established that there is no true causal effect but the same confounding structure. Besides detecting whether or not there is residual bias, there are also statistical methodologies that can be applied to then correct for this bias.^33^

#### The importance of diversity of thought

While the explicit combination of different types of analytical methods, data sources, and/or samples, is the major strength of triangulation, it may also pose certain difficulties. These may include lack of financial resources and time to collect new data, constraints in obtaining access to the required secondary data, insufficient methodological expertise, and/or insufficient cross-cultural knowledge in your research team. Some of these constraints can be counteracted by the inclusion of collaborators with relevant expertise or datasets. We recommend including diversity of thought and an interdisciplinary team approach at the idea generation stage. Incorporating broad study designs across different fields and comparing across cultural contexts are effective ways to achieve triangulation.^34^

#### Pre-registration

Finally, we highly encourage that an analysis plan is pre-registered prior to conducting analyses. Pre-registration is crucial for the validity of any study, including studies that use secondary data analysis^35^, because it will reduce questionable research practices and the effects of processes such as ‘confirmation bias’ and ‘hindsight bias’.^36^ For a triangulation study, the most crucial components to formulate and pre-register are the initial causal question, the more refined causal questions per method, the key sources of bias for each method and their expected (direction of) effect, and finally, the expected results under a true causal effect and under no causal effect. Pre-registrations can be posted on platforms such as the Open Science Framework, or researchers could consider submitting to a journal which invites registered reports.^37^

## Conclusion

In the current era of big data, triangulation will become increasingly important to be able to draw definitive conclusions about causality. With more and more large-scale (publicly) available datasets and biobanks, as well as novel sophisticated (causal) methods, there will likely be a massive influx of ‘single studies’ that make use of one dataset and apply one particular research method. If there are many published studies based on the same dataset, with similar (but not the same) applied methodologies, and these are not integrated as in a triangulation framework, then the collective knowledge base may become ‘cluttered’, taking longer to achieve a scientific consensus. Planning and conducting a triangulation study inevitably requires more time and effort than a paper comprising a single approach, resulting in a trade-off between the benefits for the individual researcher versus the benefits for the scientific field. However, with epidemiology experiencing a move towards more collaborative team science, and large amounts of available secondary data, we argue that triangulation is becoming more feasible (and necessary) than ever before. Furthermore, the resulting publication from a triangulation study is likely more impactful, making a more important contribution to the literature. That is why we would like to advocate for triangulation as the preferred approach for complex causal questions in epidemiological psychiatry. With this review, we hope to encourage more people to conduct *prospective* triangulation studies, but also to be more aware and explicit when comparing findings from different, already conducted studies to one another (for a good example of this see Shahab et al., 2022^38^). Triangulation stands as a robust and encompassing approach to attain causal conclusions that draw validation from a multifaceted array of perspectives.

## Data Availability

No empirical data or materials were employed for this review paper.
