# Supplementary material for: A guide for planning triangulation studies to investigate complex causal questions in behavioural and psychiatric research
Source: Epidemiol Psychiatr Sci. Author manuscript; Available in PMC 2024 Nov 13. (PMC7616800; doi:10.1017/S2045796024000623)
Supplement: Supplementary file 1 [file EMS198642-supplement-Supplementary_Information.pdf]

Supplementary Table 1. A broad overview of approaches (with regards to methods, samples or measures) that may be used in a triangulation study

Approach	Analysis type		Most notable strengths	Most notable weaknesses and sources of bias
Methodological triangulation	*Experimental*	Randomized controlled / clinical trial (RCT)[1]	Considered the 'gold standard' for causal inference, a reliable causal effect estimate can be obtained, provided that the relevant biases are mitigated.	Performance bias, detection bias, attrition bias. Usually not feasible for behavioural/psychiatric traits which take a long time to develop and have a low population prevalence. Ethical and practical restrictions.
		Experimental manipulation study[2]	Allows manipulation of a proposed, isolated mechanism across randomized groups to test a causal theory, usually works well for assessing short term effects.	Performance bias, detection bias, and lack of ecological validity.
		N-of-1 trial (randomized crossover trial within a single individual)[3]	Participants provide their own control prior to the introduction of an intervention/exposure. An individual is a perfect control for themselves, increasing power. Interventions can be repeatedly applied and removed to test if effects are specific. Works well for short-term effects.	Only applicable if short-term effects, which can easily be measured, are expected.
	Observational	Correction for confounders	Bias from relevant confounders is corrected for by including them as covariates in an analysis. A convenient, feasible, and flexible approach where relevant measures are available. Stratification can be performed for potential effect modifiers.	Residual confounding, reverse causation, collider bias when correcting for a variable that is not a true confounder.[4] Acyclic Graphs (DAGs) may be useful to identify true confounders (based on prior knowledge).
		Panel data (random intercept cross-lagged panel models)[5]	Allows testing of 'within subject effects' by looking at how the exposure and outcome relate to one another over time, while correcting for individual differences and changes across time for both variables separately.	Residual confounding, reverse causation, attrition bias, requires longitudinal data at preferably 3 or more time-points.
		Negative control (for either exposure or outcome)[6]	By testing the association with an exposure or outcome that is known to have no causal relation, the degree of confounding can be identified (and potentially quantified).	Must be an exposure/outcome conceivable which does not represent a causal pathway, but which is associated with similar confounding structures.
		Positive control (for either exposure or outcome)[7]	Provides a sensitivity test, by ensuring that the analysis shows evidence for a causal effect when there is a true causal effect. Helpful to identify methodological problems or biases.	Must be an available exposure/outcome which has a known true causal effect.
		Propensity score matching[8]	Estimates a casual effect without randomisation by mimicking a control group matched to exposed individuals on key characteristics. A convenient, feasible, and flexible approach.	Residual confounding, reverse causation, collider bias when correcting for a variable that is not a true confounder.[4]
		Target trial emulation[9]	After articulating the protocol of a hypothetical randomized trial (including factors such as eligibility criteria, treatment strategies, assignment, follow-up duration, outcomes), observational data are used and manipulated to emulate components of that trial.	Bias due to any element of the hypothetical trial that cannot be emulated (e.g. blinding). May not be feasible depending on the causal hypothesis and whether or not it is possible to emulate a randomized trial.
		Regression discontinuity[10]	Makes use of an intervention, environmental influence, or other abrupt change in a population (e.g. policy change) to see how that change impacts different groups ('before and after design').	Residual confounding if the probability of experiencing or being affected by the change is related to confounding factors. Only feasible if a change has occurred in a certain population that is meaningful for the causal hypothesis.
		Interrupted time series[11]	A 'before and after design', this approach assesses changes in the outcome of interest on multiple time points before and after an imposed interruption. The more time points you have, the more robust the model will be.	Residual confounding if the probability of experiencing or being affected by the change is related to confounding factors. Only feasible if an interruption has occurred or can be imposed that is meaningful for the causal hypothesis and multiple time points before and after that interruption are available.
		Difference in differences[12]	Using longitudinal panel data from an exposed and unexposed group, this approach estimates the causal effect by comparing the average longitudinal change in the outcome between the individuals in the exposed and unexposed group.	Residual confounding if the probability of experiencing or being affected by the change is related to confounding factors. Only feasible if panel data (in the same individuals) is available for multiple (>3) time-points at similar intervals around the exposure in question

	Synthetic controls[13]	By comparing an affected/treatment group to a weighted combination of groups used as a synthetic control, this method is able to predict what the data would have looked like without a certain influence (e.g. a policy change or a treatment).	The results are only reliable if the synthetic control is built from a pool of potential controls that are similar to the affected/treatment group.
	One sample Mendelian randomization (based on individual level genetic data)[14]	Because genetic variants are passed on from parents to offspring randomly (Mendel's law of segregation), using genetic variants as instrumental variables for the exposure can circumvent bias from confounding and reverse causality. Using individual level data allows flexibility to test effects specific to certain age groups or critical windows.	Horizontal pleiotropy, assortative mating, population stratification, dynastic effects. Maybe difficult to find suitably large samples with measures of the exposure, outcome and genotype data available, particularly when the exposure and/or outcome have a low prevalence.
	Two sample Mendelian randomization (based on summary level genetic data)[14]	Because genetic variants are passed on from parents to offspring randomly (Mendel's law of segregation), using genetic variants as instrumental variables for the exposure can circumvent bias from confounding and reverse causality. Very convenient as GWAS summary level data are publicly available for a large number of traits. Using summary level genetic data enables the use of a very wide range of sophisticated MR sensitivity analyses.	Horizontal pleiotropy, assortative mating, population stratification, dynastic effects, assumption that the genetic variants also predict the exposure in the outcome GWAS sample. Using genetic data as instrumental variables means that the exposure reflects a lifetime effect.
	Within-family Mendelian randomisation[15]	By conducting Mendelian randomization in family samples, bias from assortative mating, population stratification and dynastic effects can be reduced.	Horizontal pleiotropy. Difficult to find suitably large, genotyped family samples with relevant measures, particularly when the exposure and/or outcome have a low prevalence. Using genetic data as instrumental variables means that the exposure reflects a lifetime effect.
	Twin / sibling studies[16]	Because siblings and twins share their family environment and (50%/100% respectively of) their genetic material, their data can be used to test causal effects, such as with the direction of causation bivariate twin model or the discordant twin/sibling design.	Can still be confounded by non-shared environments. May be difficult to find pairs of twins/siblings with differing levels of the exposure.
	Adopted / non-adopted comparison[17]	By comparing the resemblance of adopted children to their adoptive parents and/or their birth parents, a better estimate of direct genetic influences versus shared environmental influences and dynastic effects can be obtained,	Has low practical feasibility for many causal hypotheses, as data of adopted individuals, and ideally both their birth and adoptive parents, is needed for all variables in question.
<u>Sample triangulation</u>	Cross-cohort[18]	By demonstrating a (potentially causal) association across cohorts with different selection/recruitment criteria, more reliable evidence for a causal effect can be obtained.	While easier to detect selection and confounding bias, all other biases that are relevant for the individual cohort and analytical approach still apply. May be difficult to find cohorts that are comparable enough but have different selection biases and/or confounding structures.
	Cross-age cohort[18]	By demonstrating a (potentially causal) association across cohorts from different age groups and therefore different generations, it is more likely that it is in fact a true causal effect.	While, easier to detect selection and confounding bias, all other biases that are relevant for the individual cohort and analytical approach still apply. May be difficult to find cohorts that are comparable enough but are born in different generations.
	Cross-cultural/ancestry[18]	By demonstrating a (potentially causal) association across cohorts from different cultures/ancestries with different underlying cofounding structure, it is much more likely that it is in fact a true causal effect.	While, easier to detect selection and confounding bias, all other biases that are relevant for each individual cohort and analytical approach still apply. May be difficult to find cohorts that are comparable enough but from cultures /populations that have different confounding structures relevant for the causal hypothesis.
<u>Measurement triangulation</u>	Instrumenting for[19]	Self-report and observed measurement data can have their biases, so inferences can be strengthened by comparing these with instrumental variables that can act as proxies for the exposure variable of interest.	Only feasible if there are suitable instrumental variables available, such as a genetic instrument (for MR), a suitable policy change (natural experiment), or intervention (RCT).
	Multi-measures[20]	If there is no gold standard measure available, then inferences can be strengthened by comparing across several measurement instruments. Each measure will be biased for different reasons.	Bias may be introduced if different measures capture different constructs. Only feasible if there are different types of measures available, such as self-report versus registry data or binary versus dose-response.

Multi-rater[21]	When different raters (e.g. teachers, parents, self) might suffer from different sources of bias, then it can help with interpretation to compare across several.	Reasons for differences across raters might not always be clear. Not very feasible, as multi-rater data is rarely available.

Note that this table is by no means meant as an exhaustive list of all causally informative research methods. We have focussed on methods that we feel are most relevant and useful for the field, combining the more standard and most commonly used methods with some more innovative (‘out of the box’) and potentially relatively unknown methods that may inspire a researcher’s triangulation studies. In addition, while we have highlighted the most important strengths and sources of bias, there are other sources of bias that are likely common to most or all methods (such as selection bias and self-reporting bias) and there may be more depending on the specific application of the method in question.

Supplementary Note: A Worked Example of Planning a Triangulation Study

Step 1. Determine the causal question

Our initial research question is: 'Does maternal tobacco smoking during pregnancy cause offspring depression?'

Step 2. Draw a directed acyclic graph

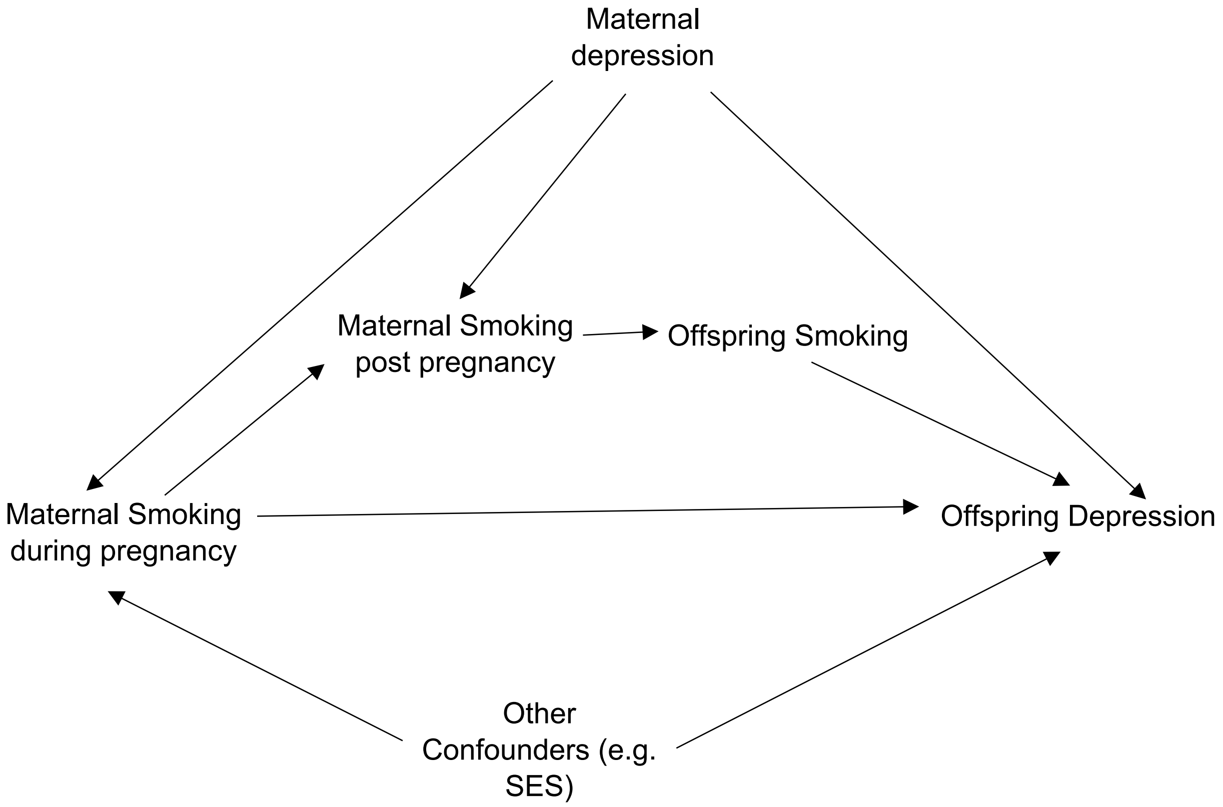

Step 3. Identify available resources and samples

Given the time lag between smoking during pregnancy and the development of offspring depression, collecting our own data is not possible for this example. Therefore, our scoping of resources focuses on examples of existing longitudinal cohorts with suitable measures which we have experience in using or for which we have collaborations through whom we might be able to obtain access. An alternative way to identify resources would be to use the Wellcome Trust's scoping report on global longitudinal resources: https://www.landscaping-longitudinal-research.com/. Furthermore, this step could include identification of different confounding structures across populations, for example, in case a population with opposite socio-economic confounding of smoking during pregnancy could be identified (although we could not think of such a context for our example).

**Study**	**Study Information and Search Tools**	**Sample Characteristics**	**Costs**	**Data**
Avon Longitudinal Study of Parents and Children (ALSPAC)	Cohort Profile Paper:<br>Fraser, A., Macdonald-Wallis, C., Tilling, K., Boyd, A., Golding, J., Davey Smith, G., ... & Lawlor, D. A. (2013). Cohort profile: the Avon Longitudinal Study of Parents and Children: ALSPAC mothers cohort. *International journal of epidemiology*, *42*(1), 97-110.<br><br>Access requirements:<br>https://www.bristol.ac.uk/alspac/researchers/access/<br><br>Data dictionary search:<br>https://variables.alspac.bris.ac.uk/	Study design: Longitudinal pregnancy cohort<br><br>Location: Avon, UK.<br><br>Sample size: 13761 mothers<br><br>Age: data from pregnancy to offspring age 30 years.<br><br>Cohort: Babies born 1990-1991.	Basic fee: £1925<br>Additional DAA: £390<br><br>Omics data: £825<br><br>(all costs +VAT where applicable)	**Smoking data:**<br>Maternal smoking during pregnancy during each trimester.<br>Paternal smoking during pregnancy.<br><br>**Measures of depression:**<br>EPDS, CCEI on mothers and partners at 11 time points.<br>SMFQ on offspring at 12 time points from 9-28 years (mixture of parent and self-report)<br><br>**Genetic data**<br>Available on parents and offspring (around ~1200 trios)
UK Biobank	Cohort Profile Paper:<br>Sudlow, C., Gallacher, J., Allen, N., Beral, V., Burton, P., Danesh, J., ... & Collins, R. (2015). UK biobank: an open access resource for identifying the causes of a wide range of complex diseases of middle and old age. *PLoS medicine*, *12*(3), e1001779.<br><br>Access requirements:	Study design: large-scale biomedical database and research resource.<br><br>Location: UK.	Tier 1: £3,000<br>Tier 2 (incl PGS): £6,000<br><br>Reduced access fees (student and ECR projects):	**Smoking data:**<br>Participant smoking (smoking status and frequency) at baseline and mental health follow-up.<br>Participant reported parental smoking around birth<br><br>**Measures of depression**:

	https://www.ukbiobank.ac.uk/enable-your-research/costs<br><br>Data dictionary search:<br>https://biobank.ndph.ox.ac.uk/showcase/search.cgi	Age: 39-72 years at recruitment.<br><br>Sample size: ~500,000<br><br>Cohort: possible birth years 1935-1971.	£500 for any tier	ICD-10 codes for depression diagnosis. Online mental health questionnaire follow-up in sub-sample.<br><br>**Genetic data**<br>Available for all study participants
Millennium Cohort Study (MCS)	Cohort Profile Paper:<br>Connelly, R., & Platt, L. (2014). Cohort profile: UK millennium cohort study (MCS). *International journal of epidemiology*, *43*(6), 1719-1725.<br><br>Access requirements:<br>https://cls.ucl.ac.uk/cls-studies/millennium-cohort-study/<br><br>Data dictionary search:<br>Questionnaire PDFs on website:<br>https://cls.ucl.ac.uk/cls-studies/millennium-cohort-study/<br>And searchable data dictionary on CLOSER:<br>https://discovery.closer.ac.uk/	Study design: Longitudinal birth cohort.<br><br>Location: UK.<br><br>Age: Birth – 24 years<br><br>Sample size: ~19,000<br><br>Cohort: Births from 2000-2002.	No cost	**Smoking data:** parent reported maternal smoking during pregnancy (reported at child 9 months) Possible to derive by trimester.<br><br>**Measures of depression:** Moods and feelings questionnaire parent-reported at age 11 years and self-reported at age 14 years.<br><br>**Genetic data:** Maternal, paternal and offspring genetic data available.
Twins Early Development Study (TEDS)	Cohort Profile Paper:<br>Haworth, C. M., Davis, O. S., & Plomin, R. (2013). Twins Early Development Study (TEDS): a genetically sensitive investigation of cognitive and behavioral development from childhood to young adulthood. *Twin Research and Human Genetics*, *16*(1), 117-125.<br><br>Access requirements:<br>https://www.teds.ac.uk/researchers/teds-data-access-policy	Study design: Longitudinal twin birth cohort.<br><br>Location: England and Wales.<br><br>Age: Birth – 30 years.	No cost	**Smoking data:** Maternal self-report of smoking during pregnancy by trimester.<br><br>**Measures of depression:** Moods and feelings questionnaire parent-reported at 12 years and self-reported at 12, 16, 21 and 26 years.

	Data dictionary:<br>https://www.teds.ac.uk/datadictionary/home.htm	Sample size: ~15,000<br><br>Cohort: Births 1994-1996		**Genetic data:** Offspring genetic data available only.
Norwegian Mother Father and Child Cohort Study (MoBa)	Cohort profile paper:<br>Magnus P, Birke C, Vejrup K, Haugan A, Alsaker E, Daltveit AK, Handal M, Haugen M, Høiseth G, Knudsen GP, Paltiel L, Schreuder P, Tambs K, Vold L, Stoltenberg C. Cohort Profile Update: The Norwegian Mother and Child Cohort Study (MoBa). Int J Epidemiol. 2016 Apr;45(2):382-8. doi: 10.1093/ije/dyw029. Epub 2016 Apr 10. PMID: 27063603.<br><br>Access requirements:<br>https://www.fhi.no/en/ch/studies/moba/for-forskere-artikler/research-and-data-access/<br><br>Data dictionary:<br>https://www.fhi.no/en/ch/studies/moba/for-forskere-artikler/questionnaires-from-moba/	Study design: Longitudinal birth cohort.<br><br>Location: Norway.<br><br>Age: Birth – 18 years.<br><br>Sample size: ~114,000 offspring, ~95,000 mothers and ~72,000 fathers<br><br>Cohort: Births from 1999-2008.	Collaborators with data access – no cost.	**Smoking data:** Maternal reported smoking during pregnancy, paternal reported smoking during pregnancy.<br><br>**Measures of depression:** sMFQ in offspring at age 8 years, 14 years, 16 years. Maternal reported depression using SCL and EPDS at 8 time points. Linkage to depression diagnoses for all participants.<br><br>**Genetic data:**<br>Available for all European ancestry participants.

Step 4. Identify suitable methodological approaches

Using supplementary Table 1 as a starting point, we identify four methods that would address the key sources of bias that we identified in step 2 by drawing a DAG, and which could be conducted using the data sources that we identified in step 3.

**Method**	**Aim**	**Description**	**Biases remaining**	**Data sources that can address this question**
Confounder adjustment	To test if there is a significant association between maternal smoking during	We will conduct a multivariate regression analysis controlling for known confounders.	Residual confounding, shared genetic liability between mother and offspring, don't know if	ALSPAC, MCS, TEDS, MoBa

	pregnancy and offspring depression after adjusting for known confounders	Known confounders include: socioeconomic position, alcohol consumption, maternal depression.	effects are solely in utero (e.g. passive smoking), collider bias (if correcting for a variable that is not a true confounder[4])	
Intergenerational Mendelian Randomisation Study	To test if there is evidence for a causal effect of maternal smoking during pregnancy on offspring depression after accounting for shared genetic liability	We will conduct a MR analysis of maternal genetic liability to smoking during pregnancy on offspring depression adjusting for child genetic liability (to account for dynastic effects) and paternal genetic liability (to prevent back door path through paternal genetics).	Horizontal pleiotropy, assortative mating, population stratification, weak instrument bias.	MoBa
Proxy GxE MR analysis	To test if genetic liability for smoking heaviness only associates with offspring depression in parents who smoked during pregnancy	Parental genotype is not available in UK Biobank so we propose to use the participants own genotype as a proxy for maternal genotype. We look for interaction effects between genetic variants associated with smoking and report of maternal smoking heaviness during pregnancy on depression in UKB participants. Analyses will also be stratified on participants own smoking status in order to estimate the	Horizontal pleiotropy, assortative mating, population stratification, dynastic effects, only proxy parental genotype (50% of effect). Timescale: using genetic data as instrumental variables means that the exposure reflects a lifetime effect.	UK Biobank

		effect of maternal smoking heaviness during pregnancy independently of offspring smoking.		
Paternal negative control analysis	To test if there is evidence for association between paternal smoking during the pregnancy and offspring depression. Is the magnitude of this association less than the association with maternal smoking (indicative of possible intrauterine effects)?	Attempt falsification using a negative control analysis. Repeat the confounder adjustment analysis and the intergenerational MR analysis using paternal smoking during pregnancy instead of maternal. Compare the magnitude of maternal and paternal associations. If they are the same magnitude then unlikely causal intrauterine effect.	Confounding structure must be the same between maternal and paternal smoking and offspring depression. If not, then not a true negative control.	ALSPAC, MoBa

Step 5: Further specify the causal question per method

The causal question that we initially posed was: ‘Does maternal tobacco smoking during pregnancy cause offspring depression?’. Now that we have chosen four methods, we define the specific causal questions for each of those methods with their accompanying samples as follows:

Method 1 (confounder adjustment): ‘In a sample of offspring born in the early 90s in Avon (UK), is mother-reported maternal tobacco smoking during pregnancy (in any trimester) associated with offspring-reported offspring depressive symptoms up to age 30 years, after adjustment for measured confounders?’

Method 2 (intergenerational MR): ‘In a sample of offspring born between 1999 and 2008 in Norway, is maternal genetic liability to smoking during pregnancy associated with offspring diagnosis of depressive disorder from patients records up to age 18 years, after offspring genetic liability and paternal genetic liability are corrected for?’

Method 3 (proxy GxE MR):’In a sample of individuals from the UK who are aged 40 years and over, is the genetic liability to heaviness of smoking of their mother (which is proxied by the individual’s own genetic risk) associated with their own risk of depression, when comparing a group of

individuals whose mothers actually smoked around the time of their birth compared to a group of individuals whose mothers didn't smoke around the time of their birth.'

Method 4a (paternal negative control analysis, confounder adjustment): 'In a sample of offspring born in the early 90s in Avon (UK), is paternal-reported paternal tobacco smoking during pregnancy (in any trimester) associated with offspring-reported offspring depressive symptoms up to age 30 years, after adjustment for measured confounders?'

Method 4b (paternal negative control analysis, intergenerational MR): 'In a sample of offspring born between 1999 and 2008 in Norway, is paternal genetic liability to smoking during pregnancy of the mother associated with offspring diagnosis of depressive disorder from patients records up to age 18 years, after offspring genetic liability and maternal genetic liability are corrected for?'

Step 6: Explicate the effects of potential biases

In this next step, we narrow down to the four methods we have chosen as well as the specific samples we would want to conduct these analyses in. This means that we can now be more specific about the specific sources of biases for each method, as well as whether each source of bias is likely to pull the estimate away from or towards the null (if known). In the following table we denote bias away from the null as '+', towards the null as '–' and '?' when direction of bias is unknown or when it could plausibly go into both directions. Note that for our worked example, there weren't many opportunities to triangulate with methods or samples that had strongly contrasting *directions* of bias (e.g. due to different confounding structures), so our framework and combination of methods relies mostly on having different *sources* of bias across the methods and samples.

**Study and sample**	**Measures**	**Biases addressed**	**Biases of method**	**Direction of bias**	**Biases of sample**	**Direction of bias**
Confounder adjustment in the ALSPAC cohort	**Exposure:** maternal smoking during any stage of pregnancy, trimester 1, trimester 2, and trimester 3. **Outcome:** offspring depressive symptoms up to 30 years.	Adjusting for bias from known confounders. Longitudinal design mitigates reverse causation, (offspring depression can't cause maternal smoking).	Residual confounding from unknown or unmeasured confounders	**+**	Attrition – less likely to participate when depressed	**-**
			Doesn't account for shared genetic	**+**	Selection – higher SES individuals	**?***

	**Confounders:** SES group at pregnancy, maternal experienced stress during pregnancy, maternal depression during pregnancy, maternal alcohol consumption.		liability to smoking.		are more likely to take part (less likely to be smokers)	
			Not specific to intrauterine exposure (e.g. could be passive smoking after birth)	**+**		
			Collider bias when correcting for a variable that is not a true confounder.[4]	**?***		
Intergenerational Mendelian randomisation in the MoBa cohort.	**Genetic instrument:** Nicotinic receptor SNP from the CHRNA5-A3-B4 gene cluster associated with smoking heaviness.<br><br>**Exposure:** maternal reported smoking during pregnancy.<br><br>**Outcome:** Any offspring diagnosis of depression from medical records.	Genetic instruments reduce bias from residual confounding and reverse causation. Testing effects in non-smokers can help to explore pleiotropy.<br>MoBa are a less selected sample, a high proportion of mothers agreed to take part.	Horizontal pleiotropy (although testing for presence using non-smokers)	**+**	Offspring up to age 18 years, before average age of depression onset	**-**
			Assortative mating	**+**	Smoking during pregnancy uncommon in this sample.	**?**
			Population stratification	**+**		
			Weak instrument bias	**+**		
Proxy GxE MR in the UK Biobank	**Genetic instrument:** Nicotinic receptor SNP from the CHRNA5-A3-	Genetic instruments reduce bias from residual confounding and reverse	Horizontal pleiotropy	**+**	Selection – UKBB is a highly	**?**

	B4 gene cluster associated with smoking heaviness.<br><br>**Exposure:**<br>smoking of the mothers of the UKB participants around birth (proxied by the participants' own genotype)<br><br>**Outcome**: ICD-10 code for diagnosis of depression or self-reported depression in the UKB participants	causation. Interaction tests for whether effects are specific to maternal smoking during pregnancy. Analyses are stratified on participants own smoking status in order to estimate the effect of maternal smoking heaviness during pregnancy independently of offspring smoking.			selected sample, high SES.	
			Assortative mating	**+**		
			Population stratification	**+**		
			Dynastic effects	**+**		
			Only proxy parental genotype (50% of effect).	**-**		
Negative control Confounder adjustment in the ALSPAC cohort	**Exposure:** paternal smoking during any stage of pregnancy, trimester 1, trimester 2, and trimester 3.<br>**Outcome:** offspring depressive symptoms up to 30 years.<br>**Confounders:** SES group at pregnancy, maternal depression during pregnancy, maternal experienced stress during pregnancy, maternal alcohol consumption during pregnancy.	Biases are the same as in the mothers, but here our expectation is that the paternal effect is significantly less than the maternal effect. The difference between the maternal and paternal effect can be used to quantify how much of the maternal effect is likely due to confounding.	Biases are the same as for the confounder adjusted analysis in mothers, listed above.			

Negative control intergenerational MR in the MoBa cohort	**Genetic instrument:** Nicotinic receptor SNP from the CHRNA5-A3-B4 gene cluster associated with smoking heaviness.<br><br>**Exposure:** paternal reported smoking during pregnancy.<br><br>**Outcome:** Any offspring diagnosis of depression from medical records.	Biases are the same as in the mothers, but here our expectation is that the paternal effect is significantly less than the maternal effect. The difference between the maternal and paternal effect can be used to quantify how much of the maternal effect is likely due to pleiotropy.	Biases are the same as for the Intergenerational Mendelian randomisation in mothers, listed above.			

* Bias in the exposure-outcome causal effect estimate could be in either direction, either masking a true effect so that it appears as null, or inducing an effect when none exists (https://mr-dictionary.mrcieu.ac.uk/term/collider-bias/).

Note that *after* the analyses have been conducted, quantitative bias analysis is a powerful approach to assess the magnitude and direction of biases.[22] Brown et al. (2024) recently provided a comprehensive overview of current methods to approximate introduced bias. Broadly, the authors introduce three methods: bias formulas, bounding methods and probabilistic bias analysis. Bias formulas offer corrected results for various types of biases. To account for unmeasured confounding, data on the prevalence of the unmeasured confounder in both the exposed and unexposed groups is required, along with its correlation with the outcome. For misclassification bias, the positive and negative predictive values for both exposure and outcome classifications are needed. When addressing selection bias, information on the likelihood of individuals being selected into the study across different exposure and outcome levels is required. Bounding methods determine an upper limit on the potential bias and require fewer assumptions about the variables involved than other techniques. Probabilistic bias analysis necessitates specifying a distribution (e.g., beta or normal) for each uncertain or estimated parameter, such as the prevalence of a confounder or the accuracy of measurements. Values are taken from these distributions and applied to bias formulas, resulting in a distribution of estimates adjusted for biases. The authors stress that the accuracy of bias approximations heavily relies on the specified expected parameters. Each method comes with its own set of assumptions and cannot completely eliminate all biases.

<u>Step 7: Pre-specify expectations under causality</u>

Finally, we consider what results would be expected under true causality and identify reasons why results may be conflicting. This step should help at the interpretation stage.

**Study**	**Timing of the exposure**	**Timing of the outcome**	**Expectations under causality**	**Expectations under non-causal**
Confounder adjustment in the ALSPAC cohort	Maternal smoking during any stage of pregnancy, trimester 1, trimester 2, and trimester 3.	Offspring self-reported depressive symptoms up to age 30 years.	Significant* association remains after adjustment for confounding.	Null association after adjusting for the confounders (original association was due to confounding).
Intergenerational MR in the MoBa cohort	Lifetime genetic liability regressed onto number of cigarettes smoked during pregnancy	Depression onset before 18 years (from diagnosis medical records so up to and including age18)	Significant association in the mothers who smoked during pregnancy and no association in the mothers who did not smoke during pregnancy.	Null association in both groups (original association was due to confounding) or significant association in both the smokers and the non-smokers (association was only due to horizontal pleiotropy).
Proxy GxE MR in UK Biobank	Lifetime genetic exposure (compared across those who do and don't smoke during pregnancy)	Lifetime diagnosis of depression from ICD-10 codes or from self-report.	Significant interaction such that the genetic variants for smoking heaviness have a larger effect for mothers who smoked during pregnancy.	Null main effect (original association was due to confounding) or no interaction effect (association unlikely through smoking during pregnancy).
Negative control Confounder adjustment in the ALSPAC cohort	Paternal smoking during any stage of pregnancy.	Offspring self-reported depressive symptoms up to age 30 years.	Significantly larger maternal effect than paternal effect.	Paternal effect and maternal effect are of the same magnitude.
Negative control intergenerational MR in the MoBa cohort	Lifetime genetic liability regressed onto number of cigarettes smoked during pregnancy	Depression onset before 18 years (from diagnosis medical records so up to and including age18)	Significantly larger maternal effect than paternal effect.	Paternal effect and maternal effect are of the same magnitude.

*As defined in your study
